# Reply to Prasad and Patankar, “Recognition of Two Distinct Pathways for Trafficking of Proteins to the Apicoplast”

**DOI:** 10.1128/mBio.03107-21

**Published:** 2021-12-21

**Authors:** Honglin Jia, Shinuo Cao, Juan Yang, Jiawen Fu

**Affiliations:** a State Key Laboratory of Veterinary Biotechnology, Harbin Veterinary Research Institute, Chinese Academy of Agricultural Sciences, Harbin, People’s Republic of China; University of Arizona

**Keywords:** apicoplast-targeting proteins, *Toxoplasma gondii*, trafficking pathway

## REPLY

In a recent study (1), we investigated the role of TgGS27 (a Qb SNARE) and TgTrs85 (a tethering factor, transport protein particle III [TRAPP III]-specific subunit) in trafficking at Golgi stacks of Toxoplasma gondii. We found that both TgGS27 and TgTrs85 are critical for intra-Golgi network trafficking. Furthermore, the proper localization of several apicoplast-residing proteins was examined in these parasite mutants, including two proteins containing both signal and transit peptides (TgCPN60 and TgACP) and others lacking a clear transit peptide (TgATrx1, TgFtsH1, and TgAPT1, the last of which lacks signal and transit peptides). We concluded that disruption of intra-Golgi network trafficking caused by the depletion of either TgGS27 or TgTrs85 impaired the proper delivery of the nucleus-encoded apicoplast-targeted (NEAT) proteins to the apicoplast. Indeed, the data based on these experiments could not provide direct evidence to tell whether these proteins were transported through the Golgi pathway because depletion of TgGS27 or TgTrs85 leads to profound effects on the function of Golgi stacks and obvious changes in the parasite morphology, as we described in the article.

We discussed the potential role of our data to support the model that the certain NEAT proteins are transported through the Golgi pathway. This speculation is based on the available information in the literature. Currently, the trafficking pathways of NEAT proteins were investigated mainly by two methods. First, the NEAT proteins fused to an ER retention signal (HDEL in T. gondii and SDEL in *Plasmodium* spp.) were examined for the proper localization in the apicoplast. Once a protein is fused with an ER retention signal, the receptor (ERD2) residing in the Golgi stacks will recognize the signal and transport the protein back to the ER through coat protein complex I (COPI)-coated vesicles. Therefore, if some NEAT proteins are trafficked through the Golgi pathway to the apicoplast, the proper delivery of these proteins will be expected to be impaired when they fuse with an ER retention signal. Based on this method, the localization of green fluorescent protein (GFP) constructs containing signal and transit sequences of apicoplast ribosomal protein S9 and acyl carrier protein (ACP) [S9(S+T)-GFP] and several other NEAT proteins (TgDer1_AP_, TgATrx1, TgATrx2, TgPPP1, andTgTic22) at the apicoplast and ER were detected by using confocal microscopy. However, the results are complicated. A couple of studies documented that the proteins were still found in apicoplasts and not observed in the ER in either T. gondii (2, 3) or Plasmodium falciparum (4), while a separate study reported that the addition of an SDEL sequence to the S9(S+T)-GFP caused reduced delivery to the apicoplast and retention in the ER in P. falciparum (5). The slight difference among these studies is that the expression of the proteins was driven by different promoters. Notice that reduced transit peptide processing of the apicoplast-targeted protein fused with an ER retention signal was observed in both studies conducted with P. falciparum (4, 5). It has been evidenced that transit peptide removal of NEAT proteins is dependent upon targeting to the apicoplast (6, 7). Therefore, reduced processing of transit peptide will reflect a slower delivery of NEAT proteins into the apicoplast. Heiny et al. suggested that the competition between the conflicting signals at Golgi stacks slowed down the transport of NEAT proteins to the apicoplast (5).

Second, in both T. gondii (2) and P. falciparum (4), the localization of the apicoplast-targeted proteins in the apicoplast was investigated by treating parasites with brefeldin A, which blocks retrograde trafficking between the ER and Golgi network by inhibiting a guanine nucleotide exchange factor (GEF) as well. Although the localization of the S9(S+T)-GFP could still be seen in the apicoplast, again, reduced processing of the transit peptide was observed in both studies. Importantly, in a brefeldin A-resistant parasite in which the sec7 domain contains a point mutation, the processing of the transit peptide did not lessen when the parasites were incubated with brefeldin A (5). These data indicated that reduced processing of the transit peptide might be caused by a lower rate of apicoplast-targeting trafficking of some NEAT proteins, which is a result of inhibition of ER-to-Golgi network trafficking by brefeldin A.

The above data seem to suggest that at least the protein carrying the signal peptide of apicoplast ribosomal protein S9 and the transit peptide of ACP are delivered through the Golgi pathway. However, more precise investigations are certainly needed to learn the trafficking pathways. The fact that a portion of NEAT proteins do not carry an obvious transit peptide suggested that multiple trafficking pathways should be involved in the traffic of NEAT proteins to the apicoplast. Probably, understanding the trafficking pathways will be better improved by identification of the sorting receptors of NEAT proteins. Another important point is that no obvious orthologue of the apicoplast-residing arginine-contributing SNARE (R-SNARE) (TgVAMP4-2) of T. gondii was found in *Plasmodium* spp. Therefore, the situations may be different in *Toxoplasma* and *Plasmodium* parasites.
